# Utilização de Dispositivo Portátil para Rastreio de Fibrilação Atrial Subclínica em Pacientes com Doença Renal Crônica Dialítica

**DOI:** 10.36660/abc.20240450

**Published:** 2025-03-27

**Authors:** Adson Patrik Vieira Carvalho, Gabriel Assis Lopes do Carmo, Cassia Aparecida Silva, Ana Cecília Oliveira, Lucas Giandoni Perez, Lilian Pires de Freitas do Carmo, Antonio L. Ribeiro

**Affiliations:** 1 Faculdade de Medicina Universidade Federal de Minas Gerais Belo Horizonte MG Brasil Faculdade de Medicina – Universidade Federal de Minas Gerais, Belo Horizonte, MG – Brasil; 2 Departamento de Cardiologia Hospital São Francisco de Assis Belo Horizonte MG Brasil Departamento de Cardiologia – Hospital São Francisco de Assis, Belo Horizonte, MG – Brasil

**Keywords:** Fibrilação atrial, Diálise, Equipamentos para Diagnóstico

## Abstract

**Fundamento:**

Os pacientes em hemodiálise (HD) apresentam altas taxas de morbidade e mortalidade cardiovascular, com maior prevalência de arritmias. A fibrilação atrial (FA) é um fator de risco independente para mortalidade e eventos tromboembólicos em pacientes em diálise. O entendimento e o manejo adequados da FA nesses pacientes dependem do conhecimento de sua prevalência. A utilização de um dispositivo portátil representaria uma abordagem pioneira para esse grupo de pacientes.

**Objetivo:**

Realizar o rastreio de pacientes em HD em relação à FA utilizando um dispositivo portátil e avaliar sua eficácia diagnóstica.

**Metodologia:**

Pacientes em HD de um hospital terciário foram submetidos ao rastreio de FA durante sessões de HD utilizando o MyDiagnostick^®^ (da fabricante Applied Biomedical Systems). Foram coletados diversos dados para avaliar as possíveis associações. A significância estatística foi definida como p < 0,05.

**Resultados:**

Foram avaliados 388 pacientes (40,7% do sexo feminino; idade média de 56,8 anos, DP ± 14,7; tempo de HD de 27 meses, 10-57). O rastreio foi positivo em 16 pacientes (4,1%). A FA foi confirmada por meio de eletrocardiograma em 7 pacientes (1,8%). Pacientes do sexo masculino (p = 0,019), em idade mais avançada (p = 0,007), com alteração no eletrocardiograma basal (p < 0,001), aumento nos níveis de potássio sérico (p = 0,021), redução da pressão arterial sistólica no início da HD (p = 0,007) e angina estável (p = 0,011) foram associados ao rastreio positivo para FA. O dispositivo apresentou uma especificidade de 91,74% (IC de 95%; 86,65% a 96,91%) e sensibilidade de 100% (IC de 95%; 100% a 100%), com um valor preditivo negativo de 100% (IC de 95%; 100% a 100%) para o rastreio da FA.

**Conclusão:**

O uso desse dispositivo se mostrou prático, com alta sensibilidade e excelente valor preditivo negativo. A FA subclínica apresenta alta prevalência e pode ser subestimada nessa população.

## Graphical abstract

**Figura Central:**
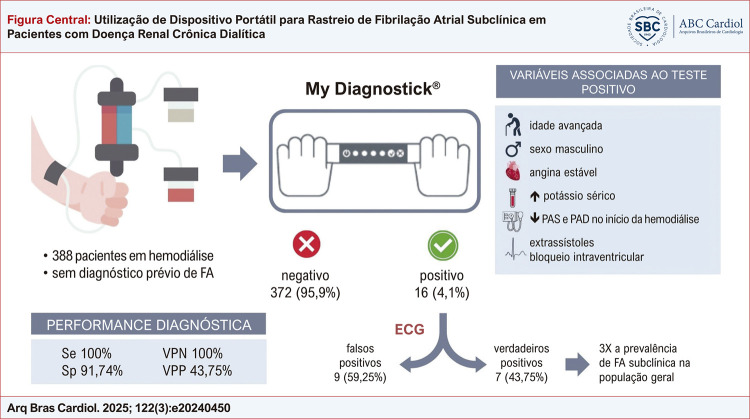
: Utilização de Dispositivo Portátil para Rastreio de Fibrilação Atrial Subclínica em Pacientes com Doença Renal Crônica Dialítica

Rastreio de fibrilação atrial subclínica em pacientes com doença renal crônica utilizando dispositivo portátil. FA: fibrilação atrial; Se: sensibilidade; Sp: especificidade; VPN: valor preditivo negativo; VPP: valor preditivo positivo; PAS: pressão arterial sistólica; PAD: pressão arterial diastólica; ECG: eletrocardiograma. Figura desenvolvida pelos autores.

## Introdução

A doença renal crônica (DRC) é um problema de saúde pública em todo o mundo, com uma prevalência estimada entre 8 e 16%.^1^ A morbidade e a mortalidade cardiovasculares estão inversamente relacionadas à taxa de filtração glomerular, com cerca de 50% de todos os óbitos entre pacientes em hemodiálise (HD) sendo atribuídos a causas cardiovasculares.^2,3^ A terapia de substituição renal (TSR) é o principal tratamento para a doença renal em estágio terminal, com a HD sendo a modalidade mais comum.^4^ Nesse grupo de pacientes, observa-se um aumento na prevalência de arritmias ventriculares, morte súbita cardíaca e fibrilação atrial (FA).^5-8^

A FA é a arritmia cardíaca mais comum na prática clínica, podendo contribuir para a redução da capacidade funcional, aumento do risco de fenômenos cardioembólicos, taxas de hospitalização, insuficiência cardíaca e morte. A prevalência global da FA é estimada entre 0,1% e 4%, com taxas crescentes nas últimas décadas.^9^ Entre os pacientes em diálise, acredita-se que a prevalência varie de 5,6% a 27%.^10,11^ A alta prevalência é parcialmente justificada pela maior ocorrência de comorbidades e aspectos específicos inerentes à TSR, como inflamação, alterações súbitas no volume sanguíneo, ativação do sistema adrenérgico e alterações nos volumes das câmaras cardíacas.^11,12^ No entanto, os dados de prevalência apresentam divergências, uma vez que há uma grande variação no desenho dos estudos e no método diagnóstico para a FA.^5,10^ Por isso, acredita-se que as FAs são subestimadas.^5,10^

Para compreender e manejar corretamente a FA em pacientes com DRC em tratamento com TSR, é essencial conhecer sua real prevalência, como ponto de partida para futuras pesquisas sobre tratamentos e complicações. O uso de um dispositivo portátil, como o MyDiagnostick^®^ (Applied Biomedical Systems, Maastricht, Países Baixos), seria pioneiro nesse grupo de pacientes e provavelmente mais eficaz do que os métodos tradicionais, já que pode ser utilizado a qualquer momento durante a HD, de modo fácil e rápido, por qualquer profissional capacitado. Portanto, o objetivo do presente estudo é avaliar a prevalência da FA subclínica entre pacientes com DRC em HD utilizando um dispositivo portátil.^13,14^

## Métodos

Trata-se de um estudo observacional com abordagem transversal que avaliou a prevalência de FA subclínica utilizando um dispositivo portátil (MyDiagnostick^®^) durante as sessões de HD nos Centros de Nefrologia do Hospital Evangélico de Belo Horizonte, além de sua acurácia diagnóstica. Este estudo foi aprovado pelo Comitê de Ética da Associação Evangélica Beneficente de Minas Gerais, de que o Hospital Evangélico faz parte, conforme os registros a seguir: Certificado de Apresentação de Apreciação Ética (CAAE) n.º 05980819.2.0000.8787 e parecer n.º 3.126.173. A avaliação da acurácia diagnóstica do dispositivo foi realizada com base no protocolo STARD (*Standards for Reporting Diagnostic Accuracy Studies*).^15^

### Pacientes

Os indivíduos foram selecionados nos Centros de Nefrologia do Hospital Evangélico de Belo Horizonte. Os critérios de inclusão foram pacientes com DRC em diálise, maiores de 18 anos, em TSR há mais de 30 dias, que concordaram em participar do estudo de forma voluntária. Foram excluídos do estudo pacientes em HD por motivos agudos e transitórios, aqueles em diálise peritoneal ou que tinham diagnóstico prévio de FA.

### Procedimentos

Os dados clínicos, sociais e epidemiológicos, comorbidades, fatores de risco cardiovascular e medicações utilizadas pelos pacientes foram retirados do prontuário clínico de cada participante. Os resultados laboratoriais referem-se aos exames realizados no mês corrente ou anteriores à coleta dos dados da pesquisa. Os dados antropométricos (peso seco, altura e índice de massa corporal) foram retirados da avaliação nutricional mais recente a que o paciente foi submetido no processo de HD. A FA clínica foi definida como a descrição da arritmia no prontuário de cada paciente, ou autodeclaração, associada à presença de um eletrocardiograma (ECG) de 12 derivações e/ou Holter compatível com o diagnóstico de FA.

Os valores de pressão arterial e ultrafiltração foram coletados no início e ao fim da sessão de HD. Os dados sobre a temperatura e o sódio da solução de diálise foram retirados do registro de HD durante o rastreio.^16,17^ Foram considerados dentro do espectro de FA subclínica os pacientes sem diagnóstico prévio, com rastreio positivo no dispositivo e com diagnóstico confirmado por ECG de 12 derivações, e que não apresentaram, durante o rastreio, sintomas de palpitações, dor no peito, dispneia, tontura, sintomas neurológicos focais ou outros sintomas frequentemente atribuídos à FA.^18^

O rastreio foi realizado na primeira sessão da semana, na primeira hora e imediatamente após o término da HD, ou seja, cada participante foi submetido ao procedimento duas vezes. Os participantes foram rastreados somente em uma sessão única. O momento escolhido para o rastreio baseou-se em estudos anteriores que demonstraram um aumento na incidência de arritmias cardíacas neste contexto específico.^12,19^ A explicação desse achado pode estar na intensidade das alterações nos eletrólitos e no volume sanguíneo que ocorrem na primeira sessão de HD da semana.^12,19^ Neste estudo, o MyDiagnostick^®^ (Applied Biomedical Systems, Maastricht, Países Baixos) foi utilizado para realizar o rastreio da FA durante as sessões de HD.^14^ Esse dispositivo conta com alta sensibilidade e especificidade, aliado ao manuseio fácil e prático.^13,14^ O MyDiagnostick^®^ é um dispositivo em forma de bastão, equipado com eletrodos sensíveis em ambas as extremidades de um cabo metálico. Ele é utilizado para analisar o ritmo cardíaco do paciente, que deve tocar e segurar o dispositivo com ambas as mãos, cada uma em uma extremidade, por um minuto. O método de detecção da FA no MyDiagnostick^®^ baseia-se na medição da irregularidade do intervalo R-R. O traço obtido é pré-processado, as ondas R são detectadas e os intervalos R-R são calculados e utilizados. O algoritmo para diagnosticar a FA leva em consideração o ritmo, a periodicidade e a variabilidade. Quando o registro for compatível com FA, uma luz vermelha se acenderá; caso não seja compatível, a luz será verde. O dispositivo oferece, ainda, uma representação gráfica que poderá ser analisada posteriormente.^14^

**Figura 1:**
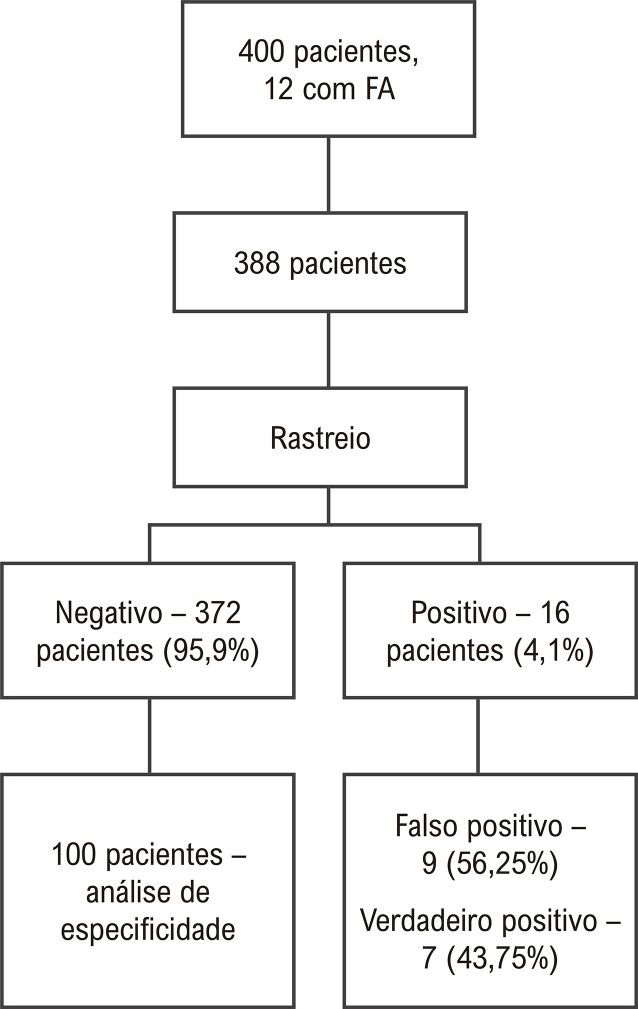
*–* Seleção de pacientes para rastreio e análise de especificidade. FA: Fibrilação atrial. Figura desenvolvida pelos autores.

Todos os registros positivos foram analisados posteriormente pelo pesquisador principal. O ECG de 12 derivações foi o método utilizado como padrão-ouro para o diagnóstico da FA. Os pacientes que tiveram rastreio positivo para FA foram submetidos ao ECG de 12 derivações para confirmação de um diagnóstico definitivo. Considerou-se como um falso positivo o participante que teve rastreio positivo para FA enquanto em uso do dispositivo portátil, mas sem confirmação simultânea do diagnóstico pelo ECG de 12 derivações. Para o diagnóstico de FA subclínica (verdadeiro positivo), foram considerados os pacientes com rastreio positivo e confirmação posterior do diagnóstico pelo ECG de 12 derivações. Uma amostra de 100 pacientes com rastreio negativo para FA e sem diagnóstico prévio de arritmia também foi submetida ao ECG de 12 derivações para confirmar a ausência de FA (Figura 1). Os ECGs foram interpretados pelo pesquisador principal.

### Análise estatística

O cálculo amostral foi realizado levando em conta um nível de confiança de 95%, prevalência de 11% da FA nos pacientes em diálise e intervalo de confiança de 8±4. A partir desses dados, estimou-se uma amostra de 235 pacientes.^20^ Considerando uma sensibilidade de 95%, IC de 10% e relação de indivíduos com rastreios negativo e positivo de 9:1, uma amostra de 100 pacientes foi submetida ao ECG de 12 derivações para realização do cálculo de especificidade.^21^

As variáveis contínuas foram apresentadas como média ± desvio padrão, quando mostraram distribuição normal, ou como mediana e intervalo interquartil, quando apresentaram distribuição assimétrica. As variáveis categóricas foram apresentadas como frequências e porcentagens. As características qualitativas foram comparadas às variáveis de resposta em tabelas de contingência utilizando o teste qui-quadrado com a correção de Yates para comparar proporções quando havia apenas duas categoriais em cada variável. O teste qui-quadrado de Pearson foi utilizado quando havia mais de duas categorias. O teste exato de Fisher foi utilizado quando pelo menos uma frequência esperada era inferior a cinco. As comparações entre as variáveis de resposta e as características na forma quantitativa foram feitas através do teste t de Student não pareado quando as suposições usuais do modelo (normalidade e homocedasticidade) foram atendidas. Caso contrário, utilizou-se o teste de Mann-Whitney. As suposições do teste t foram verificadas com base no teste de Shapiro-Wilk quanto à normalidade, e no teste de Levene, em relação à homocedasticidade. O nível de significância estatística foi definido como um valor de p < 0,05. A tabela de contingência 2x2 foi utilizada para calcular sensibilidade, especificidade, acurácia, razão de verossimilhança positiva (LR+) e valores preditivos negativo (VPN) e positivo (VPP), respectivamente. Os dados foram armazenados na plataforma REDCap e posteriormente analisados por meio do software Statistical Package for Social Sciences (SPSS), versão 20 para Windows (SPSS, Chicago, IL, EUA).^22^

## Resultados

Inicialmente, nosso estudo avaliou 400 pacientes. Nessa análise, 12 indivíduos diagnosticados com FA foram excluídos e 388 foram inscritos no estudo (Figura 1). Do total da amostra, 40,7% eram representados por pacientes do sexo feminino, com idade média de 56,8 anos (± 14,7) e mediana de tempo em HD de 27 (10-57) meses. A nefropatia diabética foi a principal etiologia encontrada, seguida pela nefroangioesclerose hipertensiva. Um histórico de síndrome coronariana aguda foi identificado em 10,6% (41) dos casos e acidente vascular cerebral, em 8,8% (34) (Tabela 1).

**Tabela 1:** – Características demográficas, clínicas, ecocardiográficas, eletrocardiográficas e laboratoriais dos pacientes

Características	População geral N = 388	Rastreio (+) N = 16	Rastreio (-) N = 372	Valor de p
Sexo feminino, N (%)	158 (40,7%)	2 (12,5%)	156 (41,9%)	0,019
Sexo masculino, N (%)	230 (59,3%)	14 (87,5%)	216 (58,1%)	
Cor, N (%)				0,633
Pardo	323 (83,2%)	12 (75%)	311 (83,8%)	
Preto	41 (10,6%)	2 (12,5%)	39 (10,5%)	
Branco	13 (3,4%)	1 (6,2%)	12 (3,2%)	
Outros	7 (1,8%)	1 (6,2%)	6 (1,6%)	
Amarelo	3 (0,8%)	0 (0%)	3 (0,8%)	
Idade (média±DP), anos	56,8 ± 14,7	66,6 ± 13	56,4 ± 14,8	0,007
Tempo de hemodiálise (mediana, IIQ), meses	27 (10,57)	36 (19,72)	27 (10,56)	0,711
Etiologia, N (%)				0,189
Nefropatia diabética	106 (27,3%)	6 (37,5%)	100 (26,9%)	
Hipertensos	98 (25,3%)	7 (43,8%)	91 (24,5%)	
Indeterminado	77 (19,8%)	2 (12,5%)	75 (20,2%)	
Outros	90 (23,2%)	1 (6,2%)	89 (23,9%)	
Glomerulopatias	17 (19,8%)	0 (0%)	17 (4,6%)	
Hipertensão, N (%)	362 (93,3%)	15 (93,8%)	347 (93,3%)	1
Diabetes mellitus NID, N (%)	33 (8,5%)	1 (6,2%)	32 (8,6%)	1
Diabetes mellitus ID, N (%)	143 (36,9%)	8 (50%)	135 (36,3%)	0,296
Dislipidemia, N (%)	49 (12,6%)	3 (18,8%)	46 (12,4%)	0,438
Tabagismo, N (%)	18 (4,6%)	0 (0%)	18 (4,8%)	1
Histórico de síndrome coronária aguda, N (%)	41 (10,6%)	1 (6,2%)	40 (10,8%)	1
Histórico de acidente vascular cerebral, N (%)	34 (8,8%)	1 (6,2%)	33 (8,9%)	1
Doença pulmonar obstrutiva crônica, N (%)	17 (4,4%)	1 (6,2%)	16 (4,3%)	0,519
Doença arterial periférica, N (%)	13 (3,4%)	2 (12,5%)	11 (3%)	0,95
Angina estável, N (%)	17 (4,4%)	1 (6,2%)	16 (4,3%)	0,011*
CCS, Classe I	13 (3,4%)	2 (12,5%)	11 (3%)	
CCS, Classe II	4 (1%)	1 (6,2%)	3 (0,8%)	
Uso de medicamentos, N (%)				
AAS	165 (42,5%)	9 (56,2%)	156 (41,9%)	0,252
Clopidogrel	14 (3,6%)	1 (6,2%)	13 (3,7%)	0,563
Varfarina	7 (1,8%)	0 (0%)	7 (1,9%)	1
Betabloqueador	232 (59,8%)	11 (68,8%)	221 (59,4%)	0,456
Bloqueador de canais de cálcio	228 (58,8%)	6 (37,5%)	222 (59,7%)	0,078
BRA	216 (55,7%)	12 (75%)	204 (54,8%)	0,112
Estatina	198 (51%)	11 (68,8%)	187 (50,3%)	0,148
Fração de ejeção (mediana, IIQ), (%)	64 (59,67)	63,5 (47,66)	64 (59,57)	0,452
Diâmetro do átrio esquerdo (mediana, IIQ), mm	42 (39,45)	45 (42,49)	42 (39,45)	0,169
ECG basal alterado, N (%)	186 (47,9%)	12 (80%)	174 (49,7%)	0,022*
Extrassístole, N (%)	11 (2,8%)	3 (18,75%)	8 (2,15%)	0,000*
Extrassístole ventricular	7 (1,8%)	2 (13,3%)	5 (1,4%)	
Extrassístole supraventricular	4 (1%)	1 (6,7%)	3 (0,9%)	
Distúrbio de condução intraventricular, N (%)	76 (19,6%)	8 (53,3%)	68 (19,4%)	0,000*
BRD	4 (1%)	0 (0%)	4 (1%)	
BRE	8 (2,1%)	1 (6,7%)	7 (2%)	
BDAS	50 (12,9%)	3 (20%)	47 (13,4%)	
BDPI	1 (0,3%)	0 (0%)	1 (0,3%)	
BRD+BDAS	6 (1,5%)	2 (13,3%)	4 (1,1%)	
BRD+ BDPI	2 (0,5%)	0 (0%)	2 (0,6%)	
Inespecífico	5 (1,3%)	2 (13,3%)	3 (0,9%)	
Hemoglobina (média±DP), g/dL	10,5 ± 2	10,5 ± 2	10,7 ± 2,1	0,754
Ureia pré-análise (média±DP), mg/dL	132 ± 43	134 ± 46,7	134 ± 43	0,963
Fósforo (mediana, IIQ), mg/dL	4,8 (3,8; 5,9)	4,5 (3; 5,7)	4,9 (3,8; 5,9)	0,205
Sódio (mediana, IIQ), mEq/L	138 (136,140)	138 (136,141)	138 (136,140)	0,859
Potássio (média±DP), mEq/L	5,5±0,9	5,5±0,9	4,9±0,9	0,021
Cálcio (mediana, IIQ), mg/dL	8,8 (8,3; 9,3)	8,5 (8,3; 9)	8,8 (8,3; 9,4)	0,335
Paratormônio (mediana, IIQ), pg/mL	306,5 (136,513)	213 (88; 349)	316 (136,520)	0,058
Ultrafiltração (mediana, IIQ), L	3 (2,5; 3,8)	2,95 (2,4; 3,5)	3 (2,5; 3,9)	0,318
PASi (mediana, IIQ), mmHg	150 (130,160)	130 (120,150)	150 (130,167)	0,007
PADi (mediana, IIQ), mmHg	80 (70,90)	80 (70,80)	80 (70,80)	0,035

Fonte: Tabela desenvolvida pelos autores. Frequência: %; média±DP: média com desvio padrão; mediana, IIQ: mediana com intervalo interquartil; NID: não dependente de insulina; ID: dependente de insulina; CCS: Sociedade Cardiovascular Canadense; AAS: ácido acetilsalicílico; ECG: eletrocardiograma; BRD: bloqueio de ramo direito; BRE: bloqueio de ramo esquerdo; BDAS: bloqueio divisional anterossuperior esquerdo; BDPI: bloqueio divisional posteroinferior esquerdo; BRA: bloqueador do receptor de angiotensina II; DAP: doença arterial periférica; PADi: pressão arterial diastólica no início da hemodiálise; PASi: pressão arterial sistólica no início da hemodiálise; *significância estatística comparada ao grupo de rastreio negativo.

### Rastreio

O rastreio foi positivo para FA em 4,1% (16) dos participantes, sendo 87,5% (14) do sexo masculino, com idade média de 66,6 ± 13 anos e mediana de tempo em HD de 36 meses. Alterações no ECG basal, com exceção da FA, estiveram presentes em 80% (12) dos pacientes.

Os pacientes do sexto masculino, com angina estável, idade avançada, presença de extrassístoles e bloqueio intraventricular no ECG basal, nível elevado de potássio e pressões arteriais sistólica e diastólica mais baixas no início da HD estavam associados ao rastreio positivo (Tabela 1).

### Desempenho do teste diagnóstico

A FA foi confirmada por meio do ECG de 12 derivações em sete pacientes que apresentaram rastreio positivo para FA, resultando em uma prevalência de FA subclínica de 1,8% nessa população. O dispositivo MyDiagnostick^®^ demostrou alta sensibilidade e especificidade, juntamente com um ótimo VPN para detecção de FA subclínica. No entanto, seu VPP foi baixo para o diagnóstico de FA subclínica (Figura Central). O teste também mostrou uma razão de verossimilhança positiva (LR+) favorável e alta acurácia geral (Tabela 2).

**Tabela 2:** – Desempenho de diagnóstico do MyDiagnostick®

ECG	Rastreio (+) N = 16	Rastreio (-) N = 100
ECG positivo	7 (43,75%)	0 (0%)
ECG negativo	9 (56,25%)	100 (100%)

Fonte: Tabela desenvolvida pelos autores. Sensibilidade = 100% (IC de 95%, 100% a 100%); especificidade = 91,74% (IC de 95%, 86,65% a 96,91%), acurácia = 92,2%, valor preditivo positivo = 43,75% (IC de 95%, 19,44% a 68%); valor preditivo negativo = 100% (IC de 95%, 100% a 100%); razão de verossimilhança positiva = 12,1 (IC de 95%, 6,5 a 22,6); ECG positivo: Eletrocardiograma de 12 derivações com um diagnóstico de FA; ECG negativo: eletrocardiograma de 12 derivações sem um diagnóstico de FA; Rastreio (+): rastreio positivo para FA utilizando o dispositivo portátil; Rastreio (-): rastreio negativo para FA utilizando o dispositivo portátil.

Os principais achados identificados como falsos positivos incluíram: batimentos ectópicos prematuros (4 casos), bloqueio sinoatrial tipo II (1 caso), ritmo atrial multifocal (1 caso), ritmo sinusal (2 casos) e tremor nas mãos (1 caso).

## Discussão

O presente estudo foi o primeiro a avaliar a prevalência da FA subclínica em uma população dialítica durante uma única sessão de HD utilizando um dispositivo portátil de rastreio. Em uma coorte de 388 pacientes, inicialmente, 4,1% dos indivíduos apresentaram rastreio positivo para FA. A confirmação posterior da FA por meio do ECG de 12 derivações estabeleceu uma prevalência da FA subclínica de 1,8%. O dispositivo MyDiagnostick^®^ demonstrou alta sensibilidade e excelente VPN, mas um VPP limitado para a detecção de FA subclínica.

A prevalência da FA subclínica encontrada em nosso estudo foi quase três vezes maior do que da população geral.^23,24^ No entanto, esse valor foi bem menor do que o encontrado em um estudo recente realizado por Al Awwa et al., que mostrou uma prevalência de 7,8% na população dialítica. Contudo, as diferenças na metodologia entre os estudos podem justificar essa divergência, como a definição da FA subclínica e o desenho do rastreio. Al Awwa et al. incluíram indivíduos com sintomas de palpitações, dor no peito, dispneia, tontura, sintomas neurológicos focais ou outros sintomas frequentemente atribuídos à FA. O rastreio foi realizado utilizando mais de um método de detecção para cada participante, incluindo o ECG de 12 variações, durante toda a sessão de HD.^25^

A alta incidência de FA na população em diálise é comumente associada à idade avançada, ao sexo masculino e ao diagnóstico prévio de doença arterial coronariana.^25-27^ Embora não seja possível estabelecer uma causalidade entre tais fatores e a FA, os participantes com um rastreio positivo para FA eram predominantemente homens, de idade mais avançada e com uma maior prevalência de angina estável.

Os distúrbios de potássio (tanto no soro pré-dialítico quanto na solução de diálise) são promissores na tentativa de compreender os gatilhos da FA.^28^ Karaboyas et al. sugerem que níveis elevados de potássio pré-diálise (> 6 mEq/L) estão associados a uma maior incidência de arritmias cardíacas.^28^ Em consonância com esses achados, o presente estudo mostrou que o rastreio positivo para FA ocorreu com maior frequência em pacientes com altos níveis de potássio em comparação com aqueles com rastreio negativo. Entretanto, é possível que o “gradiente de potássio sérico/dialisado” (diferença entre a concentração de potássio no soro e o potássio na solução de diálise) seja o fator mais relevante nesse contexto, já que os gradientes elevados levariam a uma maior variação nos níveis de potássio durante uma sessão de HD, predispondo à ocorrência de FA.

A sensibilidade e o VPN encontrados neste estudo foram semelhantes aos achados de Tieleman et al. ao estudar a população geral.^14^ Contudo, outros estudos mostraram sensibilidade e especificidade mais baixas.^29^ Em indivíduos hospitalizados com doenças cardíacas, o MyDiagnostick® demonstrou uma acurácia compatível com aquela observada neste estudo.^30^ Quando comparado a estudos que utilizaram outros dispositivos com gravação de uma única derivação, observou-se uma sensibilidade semelhante, mas uma especificidade ligeiramente mais baixa (91,7 vs. 96,5%).^31^ Entretanto, dispositivos como o MyDiagnostick^®^ são mais práticos, já que indicam a presença de rastreio positivo e são mais econômicos.^31,32^ É importante considerar que esses estudos foram realizados com populações não dialíticas, com prevalência de FA diferente. Dessa forma, a interpretação e a comparação desses achados devem ser feitas com cautela.

Este estudo avaliou uma população de alto risco cardiovascular e uma prevalência estimada de FA. O dispositivo provou ser útil para descartar a possibilidade de FA (alta sensibilidade e VPN) e aumentar a probabilidade pós-teste de apresentar FA (razão de verossimilhança positiva de 12,1). Outro achado importante foi observado entre os falsos positivos, uma vez que houve alterações eletrocardiográficas significativas em 66,6% dos casos. Se considerarmos a probabilidade de rastreio, ao identificar um ECG com alterações relevantes que possam se correlacionar com doenças cardíacas estruturais, encontramos uma especificidade de 97%, um VPP de 81,25% e uma razão de verossimilhança positiva de 34,1. Portanto, esse dispositivo teria o benefício de identificar indivíduos com outras doenças cardíacas além da FA.^33^

A impraticabilidade, a baixa disponibilidade e o alto custo de outros métodos de rastreio para FA sugerem que o MyDiagnostick^®^ pode ser utilizado de forma segura e com bom desempenho nesse cenário específico.^13,14^ Além disso, é possível que os dados coletados, junto com sensores externos e técnicas de inteligência artificial, possam contribuir na tomada de decisão tanto por parte dos pacientes quanto dos profissionais da saúde.^34,35^ Em países como o Brasil, que possuem recursos limitados na área da saúde e dificuldade de acesso a profissionais especializados, essa abordagem parece ser promissora.^30^

### Limitações

O presente estudo apresentou algumas limitações. A natureza observacional do trabalho não permite que se estabeleça uma causalidade entre as associações encontradas. A coleta de dados a partir da análise de prontuários dos pacientes pode conter vieses de registro de informações. Além disso, o rastreio realizado em uma sessão única de HD e em apenas dois momentos pode ter subestimado os resultados de nosso estudo, uma vez que a FA pode se apresentar de forma paroxística. No entanto, os rastreios com Holter de 24 horas ou dispositivos de longo prazo seriam pouco práticos e de alto custo, tornando-se inviáveis economicamente no contexto de saúde pública.

Por questões de logística, não avaliamos a reprodutibilidade teste-reteste. Da mesma forma, não foi realizada uma análise multivariada devido ao pequeno número de pacientes com FA subclínica. A confirmação do rastreio positivo ocorreu apenas com o sinal luminoso do dispositivo, sem a inspeção do registro eletrocardiográfico simultâneo, o que poderia contribuir para uma falsa redução na especificidade e no VPP. Entretanto, a inspeção manual do gráfico gerado pelo dispositivo, embora aumentasse a acurácia, tornaria inviável o rastreio no ponto de atendimento. Os nossos dados refletem uma população específica e não podem ser generalizados para outros contextos.

## Conclusões

A prevalência da FA subclínica entre pacientes com DRC em TSR durante uma única sessão de HD foi de 1,8%. O rastreio positivo para FA esteve associado a fatores como sexo masculino, idade mais avançada, angina estável, presença de extrassístoles e bloqueio intraventricular no ECG basal, níveis elevados de potássio e pressão arterial mais baixa no início da HD. O dispositivo MyDiagnostick^®^ demonstrou sensibilidade de 100%, especificidade de 91,74%, VPN de 100% e VPP de 43,75% para detecção da FA subclínica.
