# Supplementary material for: Utilização de Dispositivo Portátil para Rastreio de Fibrilação Atrial Subclínica em Pacientes com Doença Renal Crônica Dialítica
Source: Arq Bras Cardiol. 2025 Mar 27;122(3):e20240450. [Article in Portuguese] doi: 10.36660/abc.20240450 (PMC12058139; doi:10.36660/abc.20240450)
Supplement: Translation

# Translation: Subclinical Atrial Fibrillation Screening in Dialytic Chronic Kidney Disease Patients Using Portable Device

**DOI:** 10.36660/abc.20240450i

**Authors:** Adson Patrik Vieira Carvalho, Gabriel Assis Lopes do Carmo, Cassia Aparecida Silva, Ana Cecília Oliveira, Lucas Giandoni Perez, Lilian Pires de Freitas do Carmo, Antonio L. Ribeiro

**Affiliations:** 1 Brazil Faculdade de Medicina – Universidade Federal de Minas Gerais, Belo Horizonte, MG – Brazil; 2 Brazil Departamento de Cardiologia – Hospital São Francisco de Assis, Belo Horizonte, MG – Brazil

**Keywords:** Atrial Fibrillation, Dialysis, Diagnostic Equipment

## Abstract

**Background:**

Cardiovascular morbidity and mortality rates are higher in hemodialysis (HD) patients, with an increased prevalence of arrhythmias. Atrial fibrillation (AF) is an independent risk factor for mortality and thromboembolic events in dialysis patients. For a better understanding and management of AF in these patients, it is important to know its prevalence. The use of a portable device would be pioneering for this group of patients.

**Objective:**

To screen HD patients for AF using a portable gadget and evaluate the device’s diagnostic performance.

**Methods:**

HD patients at a tertiary hospital underwent AF screening during HD sessions using MyDiagnostick^®^ (Applied Biomedical Systems). Multiple data were collected to evaluate potential associations. Statistical significance was defined as p < 0.05.

**Results:**

388 patients were evaluated (female, 40.7%; mean age of 56.8 years old, SD ± 14.7; and HD time of 27 months, 10-57). Screening was positive in 16 (4.1%) patients. AF was confirmed by electrocardiogram in 7 (1.8%) patients. Male sex (p = 0.019), older age (p = 0.007), altered baseline electrocardiogram (p < 0.001), increased serum potassium (p = 0.021), reduced systolic blood pressure at the beginning of dialysis (p = 0.007), and stable angina (0.011) were associated with positive screening for AF. The device presented a 91.74% specificity (95% CI, 86.65% to 96.91%) and 100% sensitivity (95% CI, 100% to 100%), with a negative predictive value of 100% (95% CI, 100% to 100%) for AF screening.

**Conclusion:**

The use of this device proved to be practical, with high sensitivity and excellent negative predictive value. Subclinical AF has a high prevalence and may be underestimated in this population.

## Introduction

Chronic kidney disease (CKD) is a public health issue worldwide, with an estimated prevalence of between 8 and 16%.^1^ Cardiovascular morbidity and mortality have an inverse relationship with the glomerular filtration rate, and approximately 50% of all deaths among hemodialysis (HD) patients can be attributed to cardiovascular causes.^2,3^ Renal replacement therapy (RRT) is the main treatment for end-stage kidney disease, with HD being the most common modality.^4^ A higher prevalence of ventricular arrhythmias, sudden cardiac death, and atrial fibrillation (AF) can be observed in this group of patients.^5-8^

AF is the most common cardiac arrhythmia in clinical practice and may contribute to reduced functional capacity, increased risk of cardioembolic phenomena and hospitalization rates, heart failure, and death. The global prevalence of AF is estimated to be 0.1% to 4%, with a constant growth in recent decades.^9^ In the dialysis population, the prevalence is believed to be 5.6% to 27%.^10,11^ The greater occurrence of comorbidities and specific aspects inherent to RRT, such as inflammation, sudden changes in blood volume, activation of the adrenergic system, and changes in the volumes of the cardiac chambers^11,12^ partly justifies this high prevalence. Prevalence data, however, are conflicting since there is a great diversity in the design of studies and the diagnostic method for AF.^5,10^ Therefore, it is believed that AF is underestimated.^5,10^

For a better understanding and management of AF in patients with CKD undergoing RRT, it is extremely important to know its real prevalence as a starting point for future research on treatment and complications. The use of a portable device, such as MyDiagnostick^®^ (Applied Biomedical Systems, Maastricht, Netherlands), would be pioneering in this group of patients and likely more effective than traditional methods, as it can be used at any time during HD, in an easy and agile manner by any trained professional. Therefore, this study aims to evaluate the prevalence of subclinical AF among patients with CKD undergoing HD using a portable device.^13,14^

## Methods

This is a cross-sectional observational study that evaluated the prevalence of subclinical AF using a portable device (MyDiagnostick^®^) during HD sessions at the Nephrology Centers of Hospital Evangélico de Belo Horizonte and analyzed the diagnostic accuracy of that device. This study was approved by the Ethics Committee of *Associação Evangélica Beneficente de Minas Gerais,* of which the Evangelical Hospital is a part, logged under the following registrations: Certificate of Presentation of Ethical Review (CAAE) # 05980819.2.0000.8787 and opinion number 3.126.173. The assessment of the diagnostic accuracy of the device was conducted based on the STARD (“Standards for Reporting Diagnostic Accuracy Studies”) protocol.^15^

### Patients

The individuals were selected at the Nephrology Centers of Hospital Evangélico de Belo Horizonte. The inclusion criteria were patients with CKD undergoing dialysis, over 18 years of age, on RRT for over 30 days, who agreed to participate in the study voluntarily. Patients on HD for acute and transient reasons, who were on peritoneal dialysis, or who had a previous diagnosis of AF were excluded from the study.

### Procedures

Clinical, social, and epidemiological data, as well as comorbidities, cardiovascular risk factors, and medications used by the patients were extracted from each participant’s clinical record. The laboratory results refer to tests carried out in the current month or prior to the collection of research data. Anthropometric data (dry weight, height, and body mass index) were obtained from the most current nutritional assessment that the patient had undergone in the HD service. Clinical AF was defined as the description of the arrhythmia in each patient’s medical record or self-report, associated with the presence of a 12-lead electrocardiogram (ECG) and/or Holter monitoring compatible with an AF diagnosis.

Blood pressure and ultrafiltration values were collected at the beginning and end of the HD session. Data on dialysate temperature and sodium were obtained from the HD record at the time of screening.^16,17^ Patients with no prior diagnosis and with positive screening on the device, whose diagnosis was confirmed by a 12-lead ECG, and who did not present, at the time of screening, symptoms of palpitations, chest pain, dyspnea, dizziness, focal neurological symptoms, or other symptoms commonly attributed to AF were defined as having subclinical AF.^18^

The screening took place in the first session of the week, in the first hour, and immediately after the end of HD, i.e. each participant underwent screening twice. Each participant was only tracked in a single session. The moment chosen for screening was based on previous studies that demonstrated increased incidence of cardiac arrhythmias in this specific context.^12,19^ The reason for this finding may be the intensity of changes in electrolytes and blood volume that occur in the first HD session of the week.^12,19^ In this study, MyDiagnostick^®^ (Applied Biomedical Systems, Maastricht, Netherlands) was used to screen for AF during HD sessions.^14^ This device has a high sensitivity and specificity, associated with easy and practical handling.^13,14^ MyDiagnostick^®^ is a rod-shaped wand that has sensitive electrodes on both ends of a metal handle. It serves to analyze the patient’s heart rhythm by touching and holding both extremities of the device for 1 minute. The AF detection method of MyDiagnostick^®^ is based on measuring R-R interval irregularity. The trace obtained is then preprocessed, R waves are detected, and R-R intervals are calculated and used. The algorithm for diagnosing AF takes into account rhythm, periodicity, and variability. If the registration is compatible with AF, a red light appears, and if it is not compatible, a green light appears. The device also provides a graph representation that can be analyzed at a later moment.^14^

All positive records were later analyzed by the main researcher. The 12-lead ECG was the gold standard method used for diagnosing AF. Patients who screened positive for AF underwent a 12-lead ECG for definitive diagnosis. Participants who screened positive for AF using the portable device but whose diagnosis was not simultaneously confirmed by the 12-lead ECG were considered a false positive. For the diagnosis of subclinical AF (true positive), patients who screened positive for AF and whose diagnosis was subsequently confirmed by a 12-lead ECG were considered. A sample of 100 patients who screened negative for AF and with no previous diagnosis of arrhythmia also underwent a 12-lead ECG to confirm the absence of AF (Figure 1). ECGs were interpreted by the main researcher.

**Figure 1:** – Patient selection for screening and specificity analysis. AF: Atrial fibrillation. Figure prepared by the authors.

### Statistical analysis

The sample size was calculated taking into account a 95% confidence level, 11% prevalence of AF in the dialysis population, and a confidence interval of 8±4. With these data, a sample of 235 patients was estimated.^20^ Taking into account a 95% sensitivity, a 10% CI, and a 9:1 ratio of individuals who screened negative and positive, a sample of 100 individuals underwent a 12-lead ECG to calculate specificity.^21^

Continuous variables were presented as mean ± standard deviation when they showed a normal distribution, or median and interquartile range when they presented an asymmetrical distribution. Categorical variables were presented as frequency and percentage. The qualitative characteristics were compared to the response variables in contingency tables using the chi-square test with Yates correction to compare proportions when there were only two categories in each variable. If there were more than two categories, Pearson’s chi-square test was used. Fisher’s exact test was used when at least one expected frequency was less than five. Comparisons between response variables and characteristics in quantitative form were made using the Unpaired Student’s t-test when the model’s usual assumptions (normality and homoscedasticity) were met. Otherwise, the Mann-Whitney test was used. The assumptions of the t-test were verified based on the Shapiro-Wilk test for normality and the Levene test for homoscedasticity. Statistical level of significance was defined as a p-value < 0.05. To calculate sensitivity (Se), specificity (Sp), accuracy, positive likelihood ratio (LR+), and the negative and positive predictive value (NPV and PPV), the 2x2 contingency table was used. The data were stored on the REDCap platform and later analyzed using the Statistical Package for Social Sciences (SPSS) software, version 20 for Windows (SPSS, Chicago, IL, USA).^22^

## Results

Our study first evaluated 400 patients. In this analysis, 12 individuals diagnosed with AF were excluded and 388 were included in the study (Figure 1). Female individuals accounted for 40.7% of the sample, with a mean age of 56.8 years (± 14.7) and a median duration of HD of 27 (10-57) months. Diabetic nephropathy was the main etiology found, followed by hypertensive nephroangiosclerosis. A history of acute coronary syndrome was identified in 10.6% (41) of the cases, and stroke in 8.8% (34) (Table 1).

**Table 1:** – Demographic, clinical, echocardiographic, electrocardiographic, and laboratory characteristics of the patients

Characteristics	General Population N = 388	Screening (+) N = 16	Screening (-) N = 372	p-value
Female, N (%)	158 (40.7%)	2 (12.5%)	156 (41.9%)	0.019
Male, N (%)	230 (59.3%)	14 (87.5%)	216 (58.1%)	
Color, N (%)				0.633
Brown	323 (83.2%)	12 (75%)	311 (83.8%)	
Black	41 (10.6%)	2 (12.5%)	39 (10.5%)	
White	13 (3.4%)	1 (6.2%)	12 (3.2%)	
Other	7 (1.8%)	1 (6.2%)	6 (1.6%)	
Yellow	3 (0.8%)	0 (0%)	3 (0.8%)	
Age (mean ±SD), years	56.8 ± 14.7	66.6 ± 13	56.4 ± 14.8	0.007
Time of hemodialysis (median, Q1-Q3), months	27 (10.57)	36 (19.72)	27 (10.56)	0.711
Etiology, N (%)				0.189
Diabetic nephropathy	106 (27.3%)	6 (37.5%)	100 (26.9%)	
Hypertensive	98 (25.3%)	7 (43.8%)	91 (24.5%)	
Undetermined	77 (19.8%)	2 (12.5%)	75 (20.2%)	
Other	90 (23.2%)	1 (6.2%)	89 (23.9%)	
Glomerulopathies	17 (19.8%)	0 (0%)	17 (4.6%)	
Hypertension, N (%)	362 (93.3%)	15 (93.8%)	347 (93.3%)	1
Diabetes mellitus NID, N (%)	33 (8.5%)	1 (6.2%)	32 (8.6%)	1
Diabetes mellitus ID, N (%)	143 (36.9%)	8 (50%)	135 (36.3%)	0.296
Dyslipidemia, N (%)	49 (12.6%)	3 (18.8%)	46 (12.4%)	0.438
Smoking, N (%)	18 (4.6%)	0 (0%)	18 (4.8%)	1
History of Acute Coronary Syndrome, N (%)	41 (10.6%)	1 (6.2%)	40 (10.8%)	1
History of Stroke, N (%)	34 (8.8%)	1 (6.2%)	33 (8.9%)	1
Chronic Obstructive Pulmonary Disease, N (%)	17 (4.4%)	1 (6.2%)	16 (4.3%)	0.519
Peripheral Artery Disease, N (%)	13 (3.4%)	2 (12.5%)	11 (3%)	0.95
Stable angina, N (%)	17 (4.4%)	1 (6.2%)	16 (4.3%)	0.011*
CCS, Class I	13 (3.4%)	2 (12.5%)	11 (3%)	
CCS, Class II	4 (1%)	1 (6.2%)	3 (0.8%)	
Use of medication, N (%)				
AAS	165 (42.5%)	9 (56.2%)	156 (41.9%)	0.252
Clopidogrel	14 (3.6%)	1 (6.2%)	13 (3.7%)	0.563
Warfarin	7 (1.8%)	0 (0%)	7 (1.9%)	1
Betablocker	232 (59.8%)	11 (68.8%)	221 (59.4%)	0.456
Calcium channel blocker	228 (58.8%)	6 (37.5%)	222 (59.7%)	0.078
ARB	216 (55.7%)	12 (75%)	204 (54.8%)	0.112
Statin	198 (51%)	11 (68.8%)	187 (50.3%)	0.148
Ejection fraction (median, Q1-Q3), (%)	64 (59.67)	63.5 (47.66)	64 (59.57)	0.452
Diameter of the left atrium (median, Q1-Q3), mm	42 (39.45)	45 (42.49)	42 (39.45)	0.169
Altered basal ECG, N (%)	186 (47.9%)	12 (80%)	174 (49.7%)	0.022*
Extrasystole, N (%)	11 (2.8%)	3 (18.75%)	8 (2.15%)	0.000*
Ventricular Extrasystole	7 (1.8%)	2 (13.3%)	5 (1.4%)	
Supraventricular extrasystole	4 (1%)	1 (6.7%)	3 (0.9%)	
DIntraventricular flow disorder, N (%)	76 (19.6%)	8 (53.3%)	68 (19.4%)	0.000*
RBBB	4 (1%)	0 (0%)	4 (1%)	
LBBB	8 (2.1%)	1 (6.7%)	7 (2%)	
LAFB	50 (12.9%)	3 (20%)	47 (13.4%)	
LPFB	1 (0.3%)	0 (0%)	1 (0.3%)	
RBBB+LAFB	6 (1.5%)	2 (13.3%)	4 (1.1%)	
RBBB+LPFB	2 (0.5%)	0 (0%)	2 (0.6%)	
Unspecific	5 (1.3%)	2 (13.3%)	3 (0.9%)	
Hemoglobin (mean ±SD), g/dL	10.5 ± 2	10.5 ± 2	10.7 ± 2.1	0.754
Pre-analysis urea (mean ±SD), mg/dL	132 ± 43	134 ± 46.7	134 ± 43	0.963
Phosphorus (median, Q1-Q3), mg/dL	4.8 (3.8; 5.9)	4.5 (3; 5.7)	4.9 (3.8; 5.9)	0.205
Sodium (median, Q1-Q3), mEq/L	138 (136.140)	138 (136.141)	138 (136.140)	0.859
Potassium (mean ±SD), mEq/L	5.5±0.9	5.5±0.9	4.9±0.9	0.021
Calcium (median, Q1-Q3), mg/dL	8.8 (8.3; 9.3)	8.5 (8.3; 9)	8.8 (8.3; 9.4)	0.335
Parathormone (median, Q1-Q3), pg/mL	306.5 (136.513)	213 (88; 349)	316 (136.520)	0.058
Ultrafiltration (median, Q1-Q3), L	3 (2.5; 3.8)	2.95 (2.4; 3.5)	3 (2.5; 3.9)	0.318
SBPi (median, Q1-Q3), mmHg	150 (130.160)	130 (120.150)	150 (130.167)	0.007
DBPi (median, Q1-Q3), mmHg	80 (70.90)	80 (70.80)	80 (70.80)	0.035

Source: table prepared by the authors. Frequency: %; mean ± SD: mean with standard deviation; median, Q1-Q3: median with interquartile range; NID: non-insulin-dependent; ID: insulin-dependent; CCS: Canadian Cardiovascular Society; AAS: acetylsalicylic acid; ECG: electrocardiogram; RBBB: right bundle branch block; LBBB: left bundle branch block; LAFB: left anterior fascicular block; LPFB: left posterior fascicular block; ARB: angiotensin II receptor blocker; PAD: peripheral arterial disease; DBPi: diastolic blood pressure at the beginning of hemodialysis; SBPi: systolic blood pressure at the beginning of hemodialysis; *statistical significance compared to the negative screening group.

### Screening

The screening was positive for AF in 4.1% (16) of the participants; 87.5% (14) were male, mean age of 66.6 ± 13 years and a median HD time of 36 months. Changes in the baseline ECG, with the exception of AF, were present in 80% (12) of the patients.

Male sex, stable angina, high age, presence of extrasystoles and intraventricular block on the baseline ECG, high potassium level, and lower systolic and diastolic blood pressure at the beginning of HD were associated with positive screening (Table 1).

### Diagnostic test performance

AF was confirmed via a 12-lead ECG in seven patients who screened positive for AF, resulting in a 1.8% prevalence of subclinical AF in this population. MyDiagnostick^®^ demonstrated high sensitivity and specificity, along with an excellent negative predictive value (NPV) for subclinical AF detection. However, its positive predictive value (PPV) was low for the diagnosis of subclinical AF (Central Illustration). The test also exhibited a favorable positive likelihood ratio (LR+) and overall accuracy (Table 2).

**Table 2:** – Diagnostic performance of MyDiagnostick®

ECG	Screening (+) N = 16	Screening (-) N = 100
ECG positive	7 (43.75%)	0 (0%)
ECG negative	9 (56.25%)	100 (100%)

Source: table prepared by the authors. Sensitivity = 100% (95% CI, 100% to 100%), specificity = 91.74% (95% CI, 86.65% to 96.91%), accuracy = 92.2%, positive predictive value = 43.75% (95% CI, 19.44% to 68%); negative predictive value = 100% (95% CI, 100% to 100%); positive likelihood ratio = 12.1 (95% CI, 6.5 to 22.6); positive ECG: 12-lead electrocardiogram with a diagnosis of AF; negative ECG: 12-lead electrocardiogram without a diagnosis of AF; screening (+): positive screening for AF with the aid of the portable device; screening (-): negative screening for AF with the aid of the portable device.

The primary findings identified as false positives included: premature ectopic beats (4 cases), type II sinoatrial block (1 case), multifocal atrial rhythm (1 case), sinus rhythm (2 cases), and hand tremor (1 case).

## Discussion

This study is the first to assess the prevalence of subclinical AF in a dialysis population during a single HD session using a portable screening device. In a cohort of 388 patients, 4.1% initially screened positive for AF. Subsequent confirmation of AF with a 12-lead ECG established a subclinical AF prevalence of 1.8%. MyDiagnostick® demonstrated high sensitivity and an excellent NPV but a limited PPV for the detection of subclinical AF.

The prevalence of subclinical AF found in our study was almost 3-fold higher than that of the general population.^23,24^ However, this value was much lower than that found in a recent study by Al Awwa et al., which showed a 7.8% prevalence in the dialysis population. However, differences in methodology between studies may justify this discrepancy, such as the definition of subclinical AF and screening design. Al Awwa et al. included individuals with symptoms of palpitations, chest pain, dyspnea, dizziness, focal neurological symptoms, or other symptoms commonly attributed to AF. Screening was carried out using more than one detection method for each participant, including a 12-lead ECG, throughout the HD session.^25^

Advanced age, male sex, and previous diagnosis of coronary artery disease are commonly correlated with a higher incidence of AF in the dialysis population.^25-27^ Although there is no causality between such factors and AF, participants who screened positive for AF were more commonly male and older and had a higher prevalence of stable angina.

Potassium disorders (both pre-dialysis serum and dialysate) can be promising when it comes to understanding the AF triggers.^28^ Karaboyas et al. suggest that high pre-dialysis potassium levels (> 6 mEq/L) are associated with a higher incidence of cardiac arrhythmias.^28^ In line with these findings, this study showed that positive screening for AF occurred more frequently in patients with high potassium levels when compared to patients who screened negative for AF. However, the “serum/dialysate potassium gradient” (difference between the concentration of serum potassium and potassium in the dialysate) may be the most relevant factor in this context since high gradients would lead to a greater variation in potassium levels during a HD session, causing a predisposition to the occurrence of AF.

The sensitivity and NPV found in this study were similar to the findings of Tieleman et al. when studying the general population.^14^ Yet, other studies showed lower sensitivity and specificity.^29^ In hospitalized individuals with heart disease, MyDiagnostick® showed an accuracy compatible with that found in this study.^30^ When compared to studies that used other devices with a single-lead recording, we observed a similar sensitivity but a slightly lower specificity (91.7 vs 96.5%).^31^ However, devices like MyDiagnostick® are more practical, as it signals the presence of positive screening and is a more cost-effective option.^31,32^ It should be taken into account that these studies were conducted with non-dialysis populations and a different prevalence of AF. Therefore, the interpretation and comparison of these findings must be made with caution.

This study evaluated a population at a high cardiovascular risk and an estimated prevalence of AF. The device proved to be useful in ruling out the possibility of AF (high sensitivity and NPV) and increasing the post-test probability of having AF (positive likelihood ratio of 12.1). Another important finding was observed among false-positives, since there were significant electrocardiographic changes in 66.6% of the cases. If we consider the probability of the screening, when identifying an ECG with relevant changes which may correlate with structural heart disease, we will find a 97% specificity, an 81.25% PPV, and a 34.1 positive likelihood ratio. Therefore, this device would potentially detect individuals with heart disease in addition to AF.^33^

The impracticality, the reduced availability, and the high cost of other AF screening methods suggest that MyDiagnostick® can be used in this specific scenario safely and with good performance.^13,14^ Furthermore, it is possible that the data captured, associated with external sensors and artificial intelligence techniques, may play a role in decision-making by both patients and the healthcare professionals.^34,35^ In countries like Brazil, which have limited health resources and difficulty accessing specialized medical staff, this approach appears to be promising.^30^

### Limitations

This work had some limitations. The observational nature of the study does not allow one to attribute causality between the associations found. Data collection from the analysis of medical records may contain information recording biases. Furthermore, screening during a single HD session in just two moments may have underestimated the results found in our study since AF can be paroxysmal. However, 24-hour Holter screenings or long-term devices would be of little practicality and high cost and would not be economically viable in the public health scenario.

For logistic reasons, we did not evaluate test-retest reproducibility. Likewise, multivariate analysis was not performed due to the small number of patients with subclinical AF. Confirmation of the positive screening occurred only with the device’s light signal, without inspection of the simultaneous electrocardiographic recording, which could contribute to a false reduction in specificity and PPV. However, manual inspection of the graph record acquired by the device, despite increasing accuracy, would make point-of-care screening impractical. Our data reflects a specific population and cannot be generalized to other settings.

## Conclusions

The prevalence of subclinical AF among patients with CKD undergoing RRT during a single HD session was 1.8%. Positive screening for AF was associated with male sex, older age, stable angina, the presence of extrasystoles and intraventricular block on baseline ECG, elevated potassium levels, and lower blood pressure at the beginning of HD. MyDiagnostick® demonstrated a 100% sensitivity, a 91.74% specificity, a 100% NPV, as well as a 43.75% PPV for the detection of subclinical AF.
